# Genetic findings of Sanger and nanopore single-molecule sequencing in patients with X-linked hearing loss and incomplete partition type III

**DOI:** 10.1186/s13023-022-02235-7

**Published:** 2022-02-21

**Authors:** Ying Chen, Jiajun Qiu, Yingwei Wu, Huan Jia, Yi Jiang, Mengda Jiang, Zhili Wang, Hai-Bin Sheng, Lingxiang Hu, Zhihua Zhang, Zhaoyan Wang, Yun Li, Zhiwu Huang, Hao Wu

**Affiliations:** 1 Department of Otolaryngology–Head and Neck Surgery, Shanghai Ninth People’s Hospital, Shanghai Jiaotong University School of Medicine, 639 Zhizaoju Road, Shanghai, 200011 China; 2 Ear Institute, Shanghai Jiaotong University School of Medicine, Shanghai, China; 3 Shanghai Key Laboratory of Translational Medicine on Ear and Nose Diseases (14DZ2260300), Shanghai, China; 4 Department of Radiology, Ninth People’s Hospital, Shanghai Jiaotong University School of Medicine, Shanghai, China

**Keywords:** IP-III, *POU3F4*, Nanopore single-molecule sequencing, Hearing outcomes

## Abstract

**Background:**

*POU3F4* is the causative gene for X-linked deafness-2 (DFNX2), characterized by incomplete partition type III (IP-III) malformation of the inner ear. The purpose of this study was to investigate the clinical characteristics and molecular findings in IP-III patients by Sanger or nanopore single-molecule sequencing.

**Methods:**

Diagnosis of IP-III was mainly based on clinical characteristics including radiological and audiological findings. Sanger sequencing of *POU3F4* was carried out for these IP-III patients. For those patients with negative results for *POU3F4* Sanger sequencing, nanopore long-read single-molecule sequencing was used to identify the possible pathogenic variants. Hearing intervention outcomes of hearing aids (HAs) fitting and cochlear implantation (CI) were also analyzed. Aided pure tone average (PTA) was further compared between two groups of patients according to their different locations of *POU3F4* variants: in the exon region or in the upstream region.

**Results:**

In total, 18 male patients from 14 unrelated families were diagnosed with IP-III. 10 variants were identified in *POU3F4* by Sanger sequencing and 6 of these were reported for the first time (p.Gln181*, p.Val215Gly, p.Arg282Gln, p.Gln316*, c.903_912 delins TGCCA and p.Arg205del). Four different deletions that varied from 80 to 486 kb were identified 876–1503 kb upstream of *POU3F4* by nanopore long-read single-molecule sequencing. De novo genetic mutations occurred in 21.4% (3/14) of patients with *POU3F4* mutations. Among these 18 patients, 7 had bilateral HAs and 10 patients received unilateral CI. The mean aided PTA for HAs and CI users were 41.1 ± 5.18 and 40.3 ± 7.59 dB HL respectively. The mean PTAs for patients with the variants located in the exon and upstream regions were 39.6 ± 6.31 versus 43.0 ± 7.10 dB HL, which presented no significant difference (*p* = 0.342).

**Conclusions:**

Among 14 unrelated IP-III patients, 28.6% (4/14) had no definite mutation in exon region of *POU3F4*. However, possible pathogenic deletions were identified in upstream region of this gene*.* De novo genetic mutations occurred in 21.4% (3/14) of patients with *POU3F4* mutation. There was no significant difference of hearing intervention outcomes between the IP-III patients with variants located in the exon region and in the upstream region.

**Supplementary Information:**

The online version contains supplementary material available at 10.1186/s13023-022-02235-7.

## Introduction

Hearing loss is the most common sensory deficit with a prevalence of 1–2‰ in newborns [1], while genetic factors have been found to account for over 50% of cases [2]. With respect to genetic hearing loss, deafness is transmitted by an X-linked inheritance pattern in 2–3% of cases [2, 3]. To date, according to the Hereditary Hearing Loss website (http://hereditaryhearingloss.org), six loci (DFNX1–6) and five causative genes have been identified in X-linked hearing loss: *PRPS1* (OMIM 311850) for DFNX1, *POU3F4* (OMIM 300039) for DFNX2, *SMPX* (OMIM 300226) for DFNX4, *AIFM1* (OMIM 300169) for DFNX5, and *COL4L6* (OMIM 303631) for DFNX6.

*POU3F4,* encoding a transcription factor that belongs to the POU-domain family (NM_000307.4) is the most common gene for DFNX is [4, 5]. POU3F4 comprises a POU-specific domain and a POU-homeodomain, both of which influence DNA binding and specificity [6]. *POU3F4* is expressed in the developing neural tube, the supraoptic and paraventricular nuclei of the hypothalamus [5, 7], and the inner ear [8–12]. DFNX2 was originally reported in 1971 in a Caucasian kindred as an X-linked condition characterized in males by profound mixed deafness, vestibular abnormalities, and congenital fixation of the stapes [13]. In 2002, this type of cochlear abnormality was classified as incomplete partition type III (IP-III) [14]. Previous studies have identified over 90 mutations of *POU3F4* related to DFNX2 including missense/nonsense, insertions, deletions, or duplications (The Human Gene Mutation Database, http://www.hgmd.cf.ac.uk). Missense mutations in functional domains might change their structures, and would further disrupt the protein functionssuch as inducing endoplasmic reticulum stress [4, 15–17]. Frameshift truncations and extension mutations can change the stability and sub-cellular localization of the protein, which also makes them pathogenic [18].

The main phenotype of DFNX2 in male patients is severe-to-profound hearing loss accompanied with cochlear anomalies characteristic of IP-III, dilated basal turn and absent modiolus [13, 14, 19]. Cochlear implantation (CI) is one of the main methods of hearing intervention for IP-III patients although it is associated with increased risk of cerebrospinal fluid (CSF) gusher and aberrant electrode positioning in the internal auditory canal (IAC) [20]. Furthermore, CI outcomes were found to be unstable [21–23]. And the possible reason could be that those studies were based on limited case series with small sample size.

In this study, we report the clinical characteristics and molecular findings in IP-III patients by Sanger or nanopore single-molecule sequencing resulting from *POU3F4* anomalies as well as their hearing intervention outcomes.

## Materials and methods

### Patients

Between January 2017 and October 2019, 18 male patients from 14 unrelated Chinese families diagnosed with IP-III malformations were recruited in the Department of Otolaryngology-Head and Neck Surgery, Shanghai Ninth People’s Hospital Affiliated to Shanghai Jiaotong University School of Medicine. All 18 patients underwent a complete medical history inquiry and gave written, informed consent to participate in this study.

### Clinical characteristics

Auditory evaluations were conducted for each patient including otoscopic examination, tympanogram, pure tone average (PTA) or auditory brainstem responses (ABR). The degree of hearing impairment was calculated as the average of the hearing levels at 0.5, 1.0, 2.0 and 4.0 kHz for the better ear. The severity of hearing loss was classified into 4 levels: mild (26–40 dB HL), moderate (41–60 dB HL), severe (61–80 dB HL), or profound (> 80 dB HL).

Temporal bone high-resolution computed tomography (HRCT) was performed using a 64-section CT scanner (Somatom Definition Flash, Siemens Medical Solutions, Germany). Bone algorithm reconstruction was used for image acquisition. The main parameters for CT scanning were set as following: 120 kV, 150 mAs, and 0.6 mm section thickness. Cochlea, IAC, vestibule, semicircular canals, vestibular aqueduct, nerve canals in the fundus of the IAC, stapes and cochlear nerves (CN) were rigorously investigated by HRCT.

### Hearing interventions and outcome evaluations

Appropriate hearing interventions and follow-up evaluations were conducted for patients according to their age, level of hearing loss and individual factors. Aided PTA of hearing aid (HA) and CI were recorded as hearing outcomes. In cases of CI, these data were compared with 20 implantees in our center who had normal cochlear structures. The t-test was used to compare the aided PTAs using IBM SPSS Statistics, Version 19.0 (IBM Corp., Armonk, NY, USA). A *p* value less than 0.05 was considered as the threshold of statistical significance and all of the tests were two-tailed.

### Genetic analysis

Genomic DNA was extracted from blood samples using the Blood DNA kit (TIANGEN BIOTECH, Beijing, China). All probands, and other possible family members, were screened for variants of *POU3F4* coding sequences using PCR amplification and bidirectional Sanger sequencing. Possible pathogenic effects of the missense mutations were evaluated using computational tools including Mutation Taster, PROVEAN and SIFT. The allele frequencies of the detected variants detected were also investigated in 200 Chinese with normal hearing. The 3D structures of proteins were modeled using the Swiss-Model website (http://swissmodel.expasy.org) and visualized using RasMol (v. 2.7.5) software. For patients with de novo variants, paternity test by microsatellite marker was used to confirm the motherhood.

For those with negative results of *POU3F4* Sanger sequencing, nanopore long-read single-molecule sequencing was used to identify structural variants (SVs) including deletion (DEL), insertion (INS), duplication (DUP), inversion (INV), and translocation (TRA). Further annotation of SVs was performed using ANNOVAR (https://github.com/WGLab/doc-ANNOVAR) with public databases such as 1000 genome phase3, DGV gold standard CNV, dbVar nstd37, and Decipher.

## Results

### Clinical data and hearing phenotypes

**Fig. 1:**
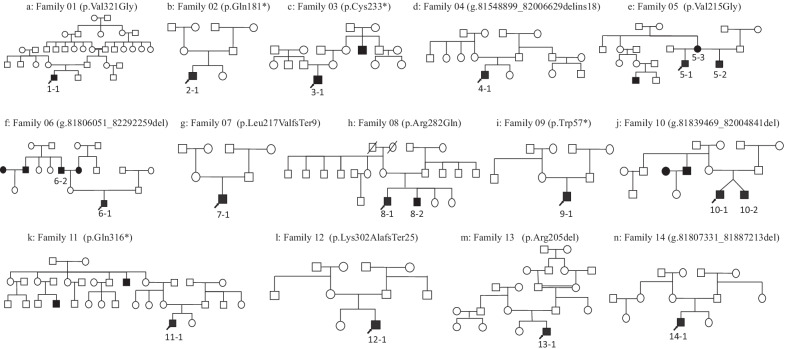
Pedigrees of the 14 unrelated families. 18 patients diagnosed with IP-III were enrolled in this study and they were numbered from 1–1 to 14–1. Patients with hearing loss were filled in shapes

In total, 18 male patients with IP-III malformations were recruited for this study (Fig. 1). The patients were from 14 unrelated Chinese families including thirteen Han and one Zang family (Family 08). The mean age at identification of hearing loss was 1.1 years (range 0.3–3.6). All patients had bilateral, prelingual, sensorineural or mixed deafness. The results of click-ABR of 14 pediatric patients ranged from 50 to > 97 dB nHL. With respect to the tympanograms, 67% (12/18) of them presented a type “A” tympanogram, 17% (3/18) had type “C”, and 11% (2/18) presented type “B”. Among them, 4 had other symptoms in addition to hearing loss (Table 1). To note, patient 4–1 was diagnosed with mild autism and had undergone bilateral tympanotomy tube insertion when he was 7 months old to treat “secretory otitis media (SOM)”.

**Table 1:** Clinical and molecular genetic characteristics of patients with IP-III malformation

Family	Patient	Age at detection (y)	Age at visit (y)	Hearing loss level^a^	Tympanogram	Other problems	Hearing intervention (side and age at which CI^b^ or HA^c^ was fitted)	*POU3F4* variants (NM_000307)
01	1–1	0.5	4.2	L 85; R 60 dB nHL	A	/	HA (L + R, 1.7y)	c.962T > G (p.Val321Gly)
02	2–1	0.4	2.4	L 80; R 70 dB nHL	A	/	CI (L, 4.0y) + HA (R, 0.7y)	c.541C > T (p.Gln181*)^e^
03	3–1	0.4	2.3	L 65; R 65 dB nHL	A		CI (R,2.7y) + HA (L,0.5y)	c.699C > A (p.Cys233*)
04	4–1	0.5	2.1	L > 97; R 80 dB nHL	C	Mild autism	CI (L,4.5y) + HA (R,2.5y)	g.81548899_82006629delins ACCAATTGGTAGTACAAT
05	5–1	0.6	3.8	L 60; R 65 dB nHL	A	/	HA (L + R, 3.8y)	c.644T > G (p.Val215Gly)^e^
	5–2	3.4	23.5	L 65; R 67.5 dB HL	A	/	HA (L + R, 14.0y)	c.644T > G (p.Val215Gly)^e^
06	6–1	0.5	0.5	L > 97; R > 97 dB nHL	A	/	CI (R, 1.0y)	g.81806051_82292259del
	6–2	3.6	67.2	L 106; R 107.5 dB HL	A	/	None	g.81806051_82292259del
07	7–1	0.5	2.0	L > 97; R > 97 dB nHL		/	CI (R, 2.5y)	c.648dupG (p.Leu217ValfsTer9)
08	8–1	3.5	14.8	L 115; R 116 dB HL	C	/	CI (R, 15.0y)	c.845G > A (p.Arg282Gln) ^e^
	8–2	3.0	12.0	L 107.5; R 105 dB HL	C	Cataract	CI (R, 12.0y)	c.845G > A (p.Arg282Gln)^e^
09	9–1	0.6	0.9	L 70; R > 97 dB nHL	A	Developmental retardation	CI (R, 1.2y) + HA (L, 0.9y)	c.171G > A (p.Trp57*)^d^
10	10–1	0.4	2.0	L > 97; R > 97 dB nHL	A	/	CI (R, 3.0y)	g.81839469_82004841del
	10–2	0.4	2.0	L > 97; R > 97 dB nHL	A	/	CI (R, 2.4y)	g.81839469_82004841del
11	11–1	0.3	0.7	L 65; R 60 dB nHL	A	/	HA (L + R, 0.7y)	c.946C > T (p.Gln316*)^e^
12	12–1	0.4	0.7	L 50; R 55 dB nHL	B	/	HA (L + R, 1.0y)	c.903_912 delins TGCCA (p.Lys302AlafsTer25)^e^
13	13–1	0.3	4.5	L 75; R 60 dB nHL	B	/	HA (L + R, 4.5y)	c.614_616delGAA (p.Arg205del)^d^
14	14–1	0.5	0.6	L 65; R 60 dB nHL	A	Atrial septal defect	HA (L + R, 0.6y)	g.81807331_81887213del^d^

^a^dB nHL: click-ABR results, dB HL: PTA results

^b^CI: cochlear implantation

^c^HA: hearing aid

^d^De novo mutation

^e^Novel variant in POU3F4 coding sequence

Among the mothers of all probands, subject 5–3 was 47 years of age and reported a history of left myringoplasty about two decades earlier. She presented mixed hearing loss in the left ear (PTA = 48.75 dB HL) and sensorineural hearing loss in the right ear (PTA = 40 dB HL). Meanwhile, HRCT scans showed that the inner ears remained unaffected.

### Radiological findings

**Fig. 2:**
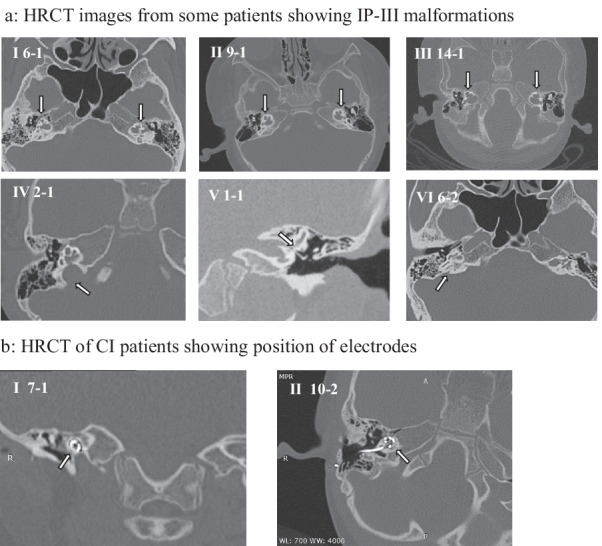
HRCT images of temporal bone and MRI of inner ear in some IP-III patients. **a** HRCT images from some patients showing IP-III malformations. I-III: “Cloudy like” characteristics of the cochlea: absence of cochlear modiolus and bony spiral lamina, and direct intercommunication between cochlea and IAC; IV: High jugular bulb; VIII-IX: Thickened stapes footplate; V: Visible vestibular aqueduct. **b** HRCT of CI patients showing position of electrodes. I: Coronal scan of patient 7–1; II: Axial scan of patient 10–2

The HRCT images showed that 18 patients had typical IP-III anomalies: bilateral and symmetrical malformation, a relatively normal shape of outer coating with absence of cochlear modiolus and bony spiral lamina. There was a direct intercommunication between cochlea and IAC with a “cloudy like” characteristic (Fig. 2a I–III). 83.3% (15/18) of patients had a high jugular bulb (Fig. 2a IV). Stapes abnormalities were present in 11.1% (2/18) of patients and included a thickened stapes footplate and absence of fissula ante fenestram (Fig. 2a V). A vestibular aqueduct was occasionally visible, with a normal shape on axial CT images (Fig. 2a VI). The semicircular canals and vestibular aqueduct shapes were also normal. Postoperative HRCT was performed in six CI users. As examples, straight electrode arrays were successfully implanted on the right side in patients 7–1 and 10–2 (Fig. 2b I–II).

### Mutations in *POU3F4* gene by Sanger sequencing

With Sanger sequencing, 12 patients from 10 families were found with hemizygous variants in exon part of *POU3F4* (Table 1) including 4 nonsense mutations, 3 missense variants, 2 frameshift mutations and one indel mutation. Apart from c.648dupG identified in Family-07, the other 6 variants were novel. Besides, two variants p.Trp57* and p.Arg205del were de novo mutations for the patients from Family-09 and -13 because their mothers had no variants of *POU3F4.* All these mutations were not present in 200 normal hearing Chinese controls (150 Han and 50 Zang).

**Fig. 3:**
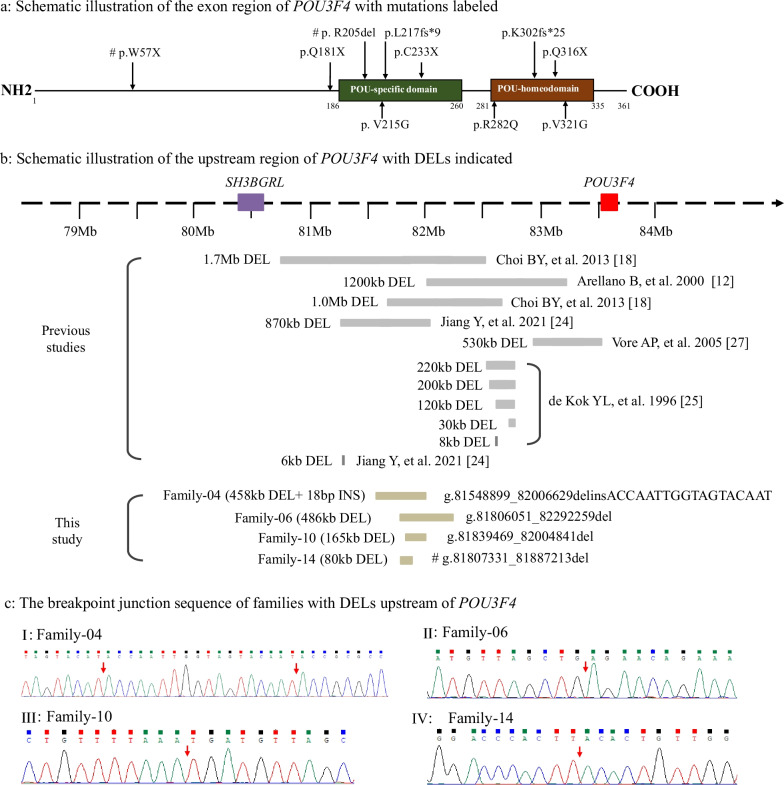
Identification of *POU3F4* variants by Sanger sequencing and nanopore long-read single-molecule sequencing. **a** Schematic illustration of the exon region of *POU3F4* with mutations labeled. Four nonsense mutations, three missense variants, two frameshift mutations and one indel mutation were identified. De novo variants are indicated with “#”. **b** Schematic illustration of the upstream region of *POU3F4* with DELs indicated. These DELs identified in this study were located between *SH3BGRL* and *POU3F4* genes on the X chromosome. Four DELs varied from 80 to 486 kb. De novo deletion is indicated with “#”. **c** The breakpoint junction sequence of families with DELs upstream of *POU3F4*. The red arrows indicate the breakpoints. An INS of 18 bp was also identified in Family-04

Four nonsense mutations, p.Gln181*, p.Cys233*, p.Trp57* and p.Gln316* were identified in Family-02, -03, -09 and -11, respectively (Fig. 3a). Mutations p.Trp57* and p.Gln181* resulted in the loss of the entire POU-specific (POUs) domain and the POU homeodomain (POU_HD_). In particular, proband 9–1 had a de novo p.Trp57* hemizygous mutation. The motherhood was confirmed by segregation of 14 short tandem repeat (STR) loci in this proband and his mother (Additional file 1: Fig. S1a). p.Cys233* was located in the POUs domain and p.Gln316* was present in the POU_HD_, both of which caused premature termination in *POU3F4*.

Three missense variants p.Val321Gly, p.Val215Gly and p.Arg282Gln were identified in Family-01, -05 and -08, respectively (Fig. 3a). These residues were also highly conserved in different species (Additional file 2: Fig. S2ab). These three variants were predicted to be disease-causing and deleterious by different prediction tools used in this study (Additional file 3: Table S1).

Two frameshift mutations c.648dupG and c.903_912 delins TGCCA were identified in Family-07 and -12, respectively. They caused amino acid mutations of p.Leu217ValfsTer9 and p.Lys302AlafsTer25, respectively (Fig. 3a), and resulted in premature termination of *POU3F4*.

The indel mutation c.614_616delGAA (p.Arg205del), identified in Family-13, was also a de novo mutation in this study. The motherhood was confirmed (Additional file 1: Fig. S1b). This residue is also highly conserved in different species (Additional file 2: Fig. S2a). The predicted structures of wild-type and mutant proteins were observed and analyzed using RasMol (Additional file 2: Fig. S2c).

### Deletions identified in upstream of *POU3F4* by nanopore long-read single-molecule sequencing

Since the results for Sanger sequencing of *POU3F4* were negative in 4 families (Family-04, -06, -10 and -14), nanopore long-read single-molecule sequencing was conducted to find possible SVs. A total of 40–50 Gb of reads with a mean length of 11.6–22 kb was obtained in each sample with an average coverage of 95.3%, and an average depth of 13–16× for the whole genome. Four different types of DELs were identified in 4 probands (Fig. 3b). The breakpoint junctions of the DELs were further verified by Sanger sequencing and the sequences of primers were listed in Additional file 4: Table S2.

In proband 4–1, we detected a 458 kb DEL and an 18 bp INS located at ChrX (g.81548899_82006629delinsACCAATTGGTAGTACAAT), approximately 1502 kb upstream of *POU3F4*. A total of 7 reads supported the DEL. The breakpoint junction and insertion were successfully sequenced in the proband and his mother (Fig. 3c I).

In proband 6–1, we identified a 486 kb DEL (g.81806051_82292259del), 1217 kb upstream of *POU3F4*. A total of 7 reads supported the DEL. The junction was successfully sequenced in the proband, his mother and his grandfather (Fig. 3c II).

In patient 10–1, a 165 kb DEL (g.81839469_82004841del) was detected, about 1503 kb upstream of the *POU3F4* gene. A total of 8 reads supported the DEL. The junction sequence was successfully amplified for the proband, his twin brother and their mother (Fig. 3c III).

In proband 14–1, we detected a 80 kb DEL (g.81807331_81887213del), 876 kb upstream of the *POU3F4* gene, and this mutation was successfully sequenced (Fig. 3c IV). His mother’s genotype was normal. The proband had a de novo DEL located upstream of *POU3F4*. The motherhood was also confirmed (Additional file 1: Fig. S1c).

### Effect of hearing intervention and its correlation with genotypes

Appropriate hearing intervention was carried out in 94.4% (17/18) of patients in this study. Ten patients received unilateral CI with or without HA on the contralateral side. Seven patients wore bilateral HAs. Only one patient did not receive hearing intervention: Patient 6–2, 67 years of age, who had congenital hearing loss, never had a HA and used sign language in daily life.

For 10 CI users, the mean aided PTA was 40.3 ± 7.59 dB HL (range 25.0–51.3 dB HL) 12 months after activation of the implant. Compared with the control group (age-matched CI recipients with normal cochlea, N = 20, PTA = 34.0 ± 5.74 dB HL), the result was not statistically different (*p* = 0.20). The genotypes of these IP-III patients consisted of 3 nonsense mutations (p.Gln181*, p.Cys233*, p.Trp57*), one frameshift mutation (c.648dupG), one missense variant (p.Arg282Gln) and 3 upstream DELs (165–486 kb).

For 7 patients with bilateral HAs, the mean aided PTA of the better ear was 41.1 ± 5.18 dB HL (range 35.0–50.0 dB HL). The genotypes of them were p.Val215Gly (two siblings), p.Val321Gly, p.Arg205del, c.903_912 delins TGCCA, p.Gln316* and g.81807331_81887213del (80 kb DEL).

Based on the location of variants, 17 patients with HAs or CI were further divided into two groups: variants located in the exon region of *POU3F4* (ER group, N = 12) or the upstream region (UR group, N = 5). The mean aided PTA of the ER group was 39.6 ± 6.31 dB HL, and that of the UR group was 43.0 ± 7.10 dB HL. The difference was not significant according to the two-tailed t-test (*p* = 0.342).

## Discussion

In this study, we analyzed the clinical characteristics, molecular variants and hearing intervention outcomes in 18 patients with IP-III from 14 unrelated families. To the best of our knowledge, it was so far the largest cohort with IP-III [24]. Ten different variants were identified in the exon region of *POU3F4*, of which six were novel. Four novel DELs in the upstream region of *POU3F4* were identified by nanopore long-read single-molecule sequencing. For the first time, this study also measured the relationship between the locations of variants in *POU3F4* and the hearing outcomes of IP-III patients.

It is a challenge to predict IP-III malformation merely from the family history and audiological outcomes during the clinical diagnosis of IP-III. In this study, 8 of 14 (57.1%) IP-III families contained sporadic cases and the level of hearing loss varied greatly. HRCT of temporal bone appears to be more reliable and is essential for IP-III diagnosis. HRCT images showed typical characteristic “cloudy like” anomalies: a relatively normal shape of outer coating, and direct intercommunication between the cochlea and IAC. Incomplete partitions constituted in 41% of inner ear malformations and IP-III occurred 2% of malformations in this region [4]. To note, HRCT examination is strongly recommended before invasive hearing intervention in children with mixed hearing loss. In this study, one patient was misdiagnosed with SOM as he did not undergo a CT scan before bilateral tympanotomy tube insertion.

In this study, Sanger sequencing detected hemizygous variants in the exon region of *POU3F4* for 71.4% (10/14) of unrelated IP-III patients. For the remaining 28.6% (4/14) patients, 80–486 kb DELs upstream of *POU3F4* were identified using nanopore long-read single-molecule sequencing. In previous studies, DELs identified upstream of *POU3F4* varied from 6 kb to 1.75 Mb [24–27]. Entire deletions, insertions and translocations of *POU3F4* were also recognized to be pathogenic in some cases [25, 28–30]. The DELs upstream of *POU3F4* were also located downstream of *SH3BGRL*, which was reported to play a role in gastric cancer, acute myeloid leukemia and breast cancer [31–33]. However, whether DELs located between *POU3F4* and *SH3BGRL* genes influence the function of *SH3BGRL* remains unknown.

It is worth mentioning that 21.4% (3/14) de novo mutations of *POU3F4* were found to include two mutations located in the exon region and one mutation in the upstream region. Motherhood was confirmed by the segregation of a few microsatellite markers for these three cases. In a previous study, de novo genetic mutations occurred in 20% of patients with *POU3F4* mutations [28]. These results indicate that the high rate of de novo mutations is one reason for the relatively high incidence of sporadic cases of *POU3F4* variants. To note, two probands with de novo mutations located in the exon region (p.Trp57* and p.Arg205del) presented relatively asymmetric hearing loss (interaural threshold gap ≥ 15 dB nHL).

Hearing intervention outcomes were reported to vary greatly among IP-III patients [16, 25]. Stankovic et al. reported limited auditory perception and language acquisition after CI in IP-III patients and commented that HA may sometimes be a better alternative than CI [23]. In our study, aided PTA in HA users and CI recipients were 41.1 ± 5.18 dB HL and 40.3 ± 7.59 dB HL, respectively. These results showed that CI was also an optimal intervention method for IP-III patients. This study, for the first time, focused on the correlation between genotype and hearing outcomes based on the variants’ locations. As a result, the aided PTA presented no difference regardless of the variants’ locations on exon or upstream regions (*p* = 0.342).

This study has three potential limitations. The first is the short follow-up period of 12 months for hearing outcomes. Long-term follow-up is still needed. The second limitation is the uncertainty over the pathogenicity of variants, especially DELs identified in the upstream region of *POU3F4.* It is necessary to carry out functional studies at the animal level to further confirm the pathogenicity of variants. The final limitation of this study is the lack of sensitive indicators to predict outcomes of hearing intervention in IP-III patients. The benefits of CI vary greatly among individual patients [23, 34, 35]. It has also been demonstrated that the number and capability of spiral ganglion neurons (SGNs) are important factors for CI outcomes [36–38]. Animal studies have shown that the deletion of Pou3f4 in the otic mesenchyme could cause the impair of the radial bundle fasciculation and hair cell innervation of SGNs [36]. According to a recent animal research, the absence of the mesenchyme-specific transcription factor Pou3f4 could cause the apoptosis of 25% of the overall SGN population after birth [39]. Whether there is a neurophysiological or other indicators which might provide clinical prediction of the function of SGNs and CI outcomes is still unknown.

In conclusion, no mutation was identified in exon region of *POU3F4* for 28.6% (4/14) of IP-III patients. However, possible pathogenic deletions were identified in upstream region of this gene*.* De novo genetic mutations occurred in 21.4% (3/14) of patients with *POU3F4* mutation. Hearing intervention outcomes of IP-III patients presented no difference regardless of the variants locations on exon or upstream regions.

## Supplementary Information

**Additional file 1: Fig. S1.** Motherhood confirmation of three families with de novo variants. 14 genetic markers (D19S433, D5S818, D18S51, D6S1043, D3S1358, D16S539, CSF1PO, VWA, TPOX, Penta E, THO1, D12S391, D2S1338, and FGA) are used to identify paternity. (**a**) Family 09 with p.Trp57*. The relative chance of paternity (RCP) is 99.9999%. (**b**) Family 13 with p.Arg205del. The RCP is 99.9999%. (**c**) Family 14 with g.81807331_81887213del. The RCP is 99.9999%.

**Additional file 2: Fig. S2.** Conservation analysis of variants located in the POU-specific domain and homeodomain of *POU3F4*. (**a**) Protein alignment of POU-specific domain across six species. The three residues (R205, V215 and L217) located in the POU-specific domain were highly conserved in these species. (**b**) Protein alignment of POU homeodomain across six species. The three residues (R282, K302 and V321) located in the POU homeodomain were also highly conserved in these species. (**c**) Structural simulation of p.Arg205del mutant protein. The 205 residues are marked in green (white arrows) and the difference in protein structures is clearly seen between mutant protein (MT) and wild-type (WT) protein.

**Additional file 1: Table S1.** Pathogenicity prediction of three missense variants and their classification according to the ACMG rules.

**Additional file 1: Table S2.** Primer sequences from PCR amplification for the exon and breakpoint junctions located in the upstream region of *POU3F4*.

## Data Availability

Please contact author for data requests.

## Abbreviations

IP-III: Incomplete partition type III
DELs: Deletions
PTA: Pure tone average
CI: Cochlear implantation
CSF: Cerebrospinal fluid
IAC: Internal auditory canal
HRCT: High-resolution computed tomography
CN: Cochlear nerves
ABR: Auditory brainstem responses
IT-MAIS: Infant–toddler meaningful auditory integration scale
CAP: Categories of auditory performance
MUSS: Meaningful use of speech scale
SIR: Speech intelligibility rating
SV: Structural variants
INS: Insertion
DUP: Duplication
INV: Inversion
TRA: Translocation
SOM: Secretory otitis media
NHS: Newborn hearing screening
CISS: Constructive interference in steady-state
HA: Hearing aid
ER: Exon region
UR: Upstream region
