# Supplementary figures and images for: Genetic findings of Sanger and nanopore single-molecule sequencing in patients with X-linked hearing loss and incomplete partition type III
Source: Orphanet J Rare Dis. 2022 Feb 21;17:65. doi: 10.1186/s13023-022-02235-7 (PMC8862311; doi:10.1186/s13023-022-02235-7)

a: Family 09 (p.Trp57\*)

Proband

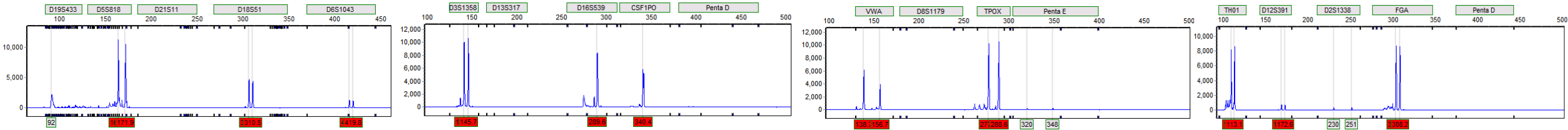

Mother

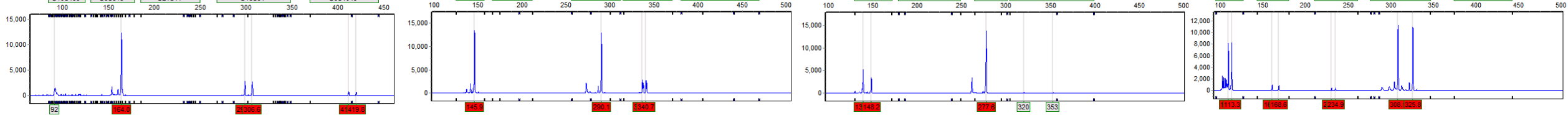

b: Family 13 (p.Arg205del)

Proband

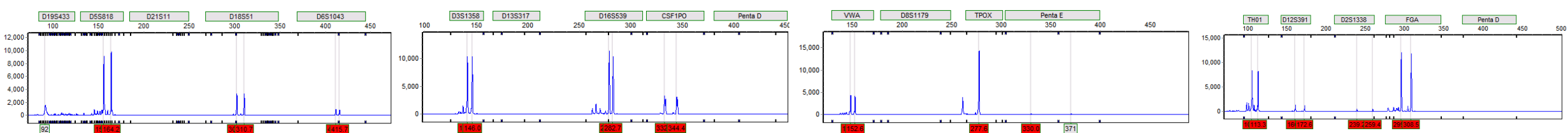

Mother

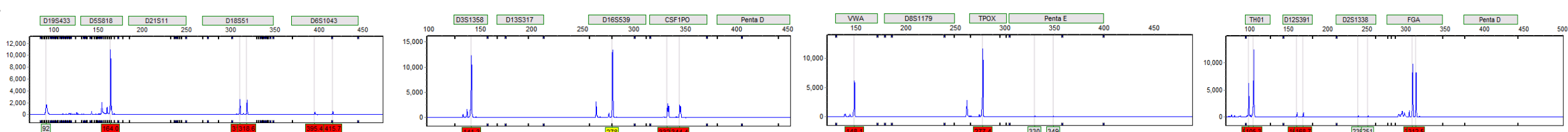

c: Family 14 (g.81807331\_81887213del)

Proband

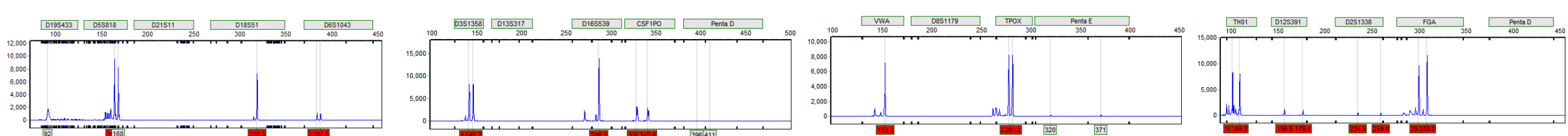

Mother

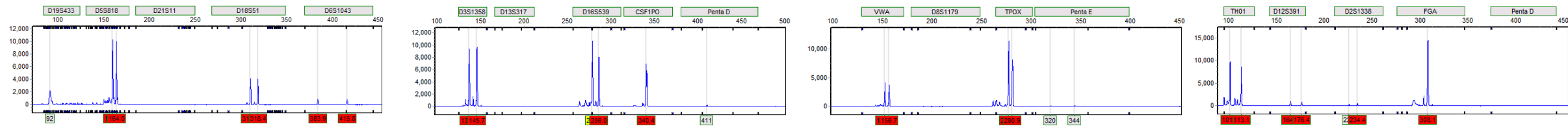

Supplement: Supplementary file 1 — Additional file 1: Fig. S1. Motherhood confirmation of three families with de novo variants. 14 genetic markers (D19S433, D5S818, D18S51, D6S1043, D3S1358, D16S539, CSF1PO, VWA, TPOX, Penta E, THO1, D12S391, D2S1338, and FGA) are used to identify paternity. (a) Family 09 with p.Trp57*. The relative chance of paternity (RCP) is 99.9999%. (b) Family 13 with p.Arg205del. The RCP is 99.9999%. (c) Family 14 with g.81807331_81887213del. The RCP is 99.9999%. [file 13023_2022_2235_MOESM1_ESM.pdf]
