# Supplementary material for: Genetic findings of Sanger and nanopore single-molecule sequencing in patients with X-linked hearing loss and incomplete partition type III
Source: Orphanet J Rare Dis. 2022 Feb 21;17:65. doi: 10.1186/s13023-022-02235-7 (PMC8862311; doi:10.1186/s13023-022-02235-7)

a: Protein alignment of POU-specific domain across six species

Residue range 186–260; boxed positions: R205, V215, L217

Species	Sequence (186–260)
**Homo sapiens**	EETPTSDELEQFAKQFKQRRIKLGFTQADVGLALGTLYGNVFSQTTICRFEALQLSFKNMCKLKPLLNKWLEEAD
**Mus musculus**	EETPTSDELEQFAKQFKQRRIKLGFTQADVGLALGTLYGNVFSQTTICRFEALQLSFKNMCKLKPLLNKWLEEAD
**Rattus norvegicus**	EETPTSDELEQFAKQFKQRRIKLGFTQADVGLALGTLYGNVFSQTTICRFEALQLSFKNMCKLKPLLNKWLEEAD
**Macaca mulatta**	EETPTSDELEQFAKQFKQRRIKLGFTQADVGLALGTLYGNVFSQTTICRFEALQLSFKNMCKLKPLLNKWLEEAD
**Gallus gallus**	EETPTSDELEQFAKQFKQRRIKLGFTQADVGLALGTLYGNVFSQTTICRFEALQLSFKNMCKLKPLLNKWLEEAD
**Xenopus tropicalis**	EETPTSDELEQFAKQFKQRRIKLGFTQADVGLALGTLYGNVFSQATICRFEALQLSFKNMCKLKPLLNKWLEEAD

b: Protein alignment of POU homeodomain across six species

Residue range 278–340; boxed positions: R282, K302, V321

Species	Sequence (278–340)
**Homo sapiens**	KRKKRTSIEVSVKGVLETHFLKCPKPAAQEISSLADSLQLEKEVVRVWFCNRRQKEKRMTPPG
**Mus musculus**	KRKKRTSIEVSVKGVLETHFLKCPKPAAQEISSLADSLQLEKEVVRVWFCNRRQKEKRMTPPG
**Rattus norvegicus**	KRKKRTSIEVSVKGVLETHFLKCPKPAAQEISSLADSLQLEKEVVRVWFCNRRQKEKRMTPPG
**Macaca mulatta**	KRKKRTSIEVSVKGVLETHFLKCPKPAAQEISSLADSLQLEKEVVRVWFCNRRQKEKRMTPPG
**Gallus gallus**	KRKKRTSIEVSVKGVLETHFLKCPKPAAQEISSLADSLQLEKEVVRVWFCNRRQKEKRMTPPG
**Xenopus tropicalis**	KRKKRTSIEVSVKGVLETHFLKCPKPAAQEISSLADSLQLEKEVVRVWFCNRRQKEKRMTPPG

c: Structural simulation of p.Arg205del mutant protein

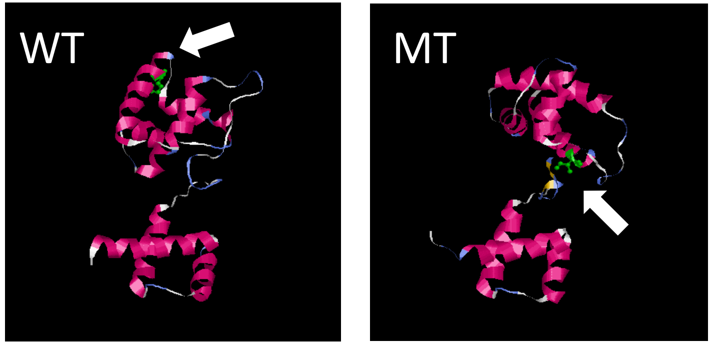

Supplement: Supplementary file 2 — Additional file 2: Fig. S2. Conservation analysis of variants located in the POU-specific domain and homeodomain of POU3F4. (a) Protein alignment of POU-specific domain across six species. The three residues (R205, V215 and L217) located in the POU-specific domain were highly conserved in these species. (b) Protein alignment of POU homeodomain across six species. The three residues (R282, K302 and V321) located in the POU homeodomain were also highly conserved in these species. (c) Structural simulation of p.Arg205del mutant protein. The 205 residues are marked in green (white arrows) and the difference in protein structures is clearly seen between mutant protein (MT) and wild-type (WT) protein. [file 13023_2022_2235_MOESM2_ESM.pdf]
