# Supplementary material for: Genetic findings of Sanger and nanopore single-molecule sequencing in patients with X-linked hearing loss and incomplete partition type III
Source: Orphanet J Rare Dis. 2022 Feb 21;17:65. doi: 10.1186/s13023-022-02235-7 (PMC8862311; doi:10.1186/s13023-022-02235-7)
Supplement: Supplementary file 3 — Additional file 1: Table S1. Pathogenicity prediction of three missense variants and their classification according to the ACMG rules. [file 13023_2022_2235_MOESM3_ESM.docx]

**Additional file 3: Table S1:** **Pathogenicity prediction of three missense variants and their** **classification according to the ACMG rules**

| Variants | Domain | Mutation taster | | PROVEAN | | SIFT | | Metadome | | ACMG |
| --- | --- | --- | --- | --- | --- | --- | --- | --- | --- | --- |
|  |  | Score | Prediction | Score | Prediction | Score | Prediction | Score | Prediction |  |
| p.Val215Gly | POU-specific domain | 4.57 | Disease causing | -7.00 | Deleterious | 0.000 | Damaging | 0.04 | Highly intolerant | PM1+PM2+PP1+PP4 |
| p.Arg282Gln | POU homeodomain | 5.23 | Disease causing | -4.00 | Deleterious | 0.000 | Damaging | 0.32 | Intolerant | PM1+PM2+PP1+PP4 |
| p.Val321Gly | POU homeodomain | 4.593 | Disease causing | -6.95 | Deleterious | 0.000 | Damaging | 0.1 | Highly intolerant | PS1+PM1+PM2+PP1+PP4 |
