# Supplementary material for: Genetic findings of Sanger and nanopore single-molecule sequencing in patients with X-linked hearing loss and incomplete partition type III
Source: Orphanet J Rare Dis. 2022 Feb 21;17:65. doi: 10.1186/s13023-022-02235-7 (PMC8862311; doi:10.1186/s13023-022-02235-7)
Supplement: Supplementary file 4 — Additional file 1: Table S2. Primer sequences from PCR amplification for the exon and breakpoint junctions located in the upstream region of POU3F4. [file 13023_2022_2235_MOESM4_ESM.docx]

**Additional file 4: Table S2: Primer sequences from PCR amplification for the exon and breakpoint junctions located in the upstream region of *POU3F4***

| Amplification location | Primer number | Sequence |
| --- | --- | --- |
| Exon-front half | Forward - 1 | GGTAACCCGTGCTAGCGTCTT |
|  | Reverse - 1 | CGGAGTGATCCTGGCAATGGT |
| Exon-latter half | Forward - 2 | ATGCTGGAACACGGGGGACTC |
|  | Reverse - 2 | GAAATCCGCGCTGCTCCCA |
| g.81548899_82006629delins18  (Family-04) | Forward - 3 | GCCACCAGTGTATACCAAACAA |
|  | Reverse - 3 | GCTAGAAATTACCATGACTCAAGACA |
| g.81806051_82292259del  (Family-06) | Forward - 4 | AATGGCCTTCTTTGTCTCTTT |
|  | Reverse - 4 | ATGTTCTTGGTGCCTTTGTTA |
| g. 81839469_82004841del  (Family-10) | Forward - 5 | AGATGACGACTTGATGGGTG |
|  | Reverse - 5 | CTAGCATTTATTGGGCATTTACT |
| g.81807331_81887213del  (Family-14) | Forward - 6 | GGTCTGTTGGTGTACCCAGCT |
|  | Reverse - 6 | CTTTATTCATTCATCTGCTAATGA |
